# The association between triglyceride-glucose index and the likelihood of cardiovascular disease in the U.S. population of older adults aged ≥ 60 years: a population-based study

**DOI:** 10.1186/s12933-024-02248-5

**Published:** 2024-05-03

**Authors:** Dan Liang, Chang Liu, Yan Wang

**Affiliations:** 1 Department of Endocrine, People’s Hospital of Chongqing Liang Jiang New Area, Chongqing, China; 2 School of Medicine, Nankai University, Tianjin, China

**Keywords:** Triglyceride-glucose index, Cardiovascular disease, Cross-section study, Population-based study, NHANES

## Abstract

**Background:**

The association between the triglyceride-glucose (TyG) index and the likelihood of developing cardiovascular disease (CVD) in the general elderly population in the United States aged 60 and above is not well understood. The objective of our study was to examine the relationship between the TyG index and CVD likelihood in the general elderly population over 60 years of age in the United States.

**Methods:**

Data for this cross-sectional study were sourced from the 2003–2018 National Health and Nutrition Examination Survey. Weighted multivariable regression analysis and subgroup analysis were conducted to estimate the independent relationship between the TyG index and the likelihood of CVD. Non-linear correlations were explored using restricted cubic splines.

**Results:**

A total of 6502 participants were included, with a mean TyG index of 8.75 ± 0.01. The average prevalence of CVD was 24.31% overall. Participants in the higher TyG quartiles showed high rates of CVD (Quartile 1: 19.91%; Quartile 2: 21.65%; Quartile 3: 23.82%; Quartile 4: 32.43%). For CVD, a possible association between the TyG index and the odds of CVD was observed. Our findings suggest a nonlinear association between the TyG index and the odds of CVD. The threshold of 8.73 for the likelihood of CVD. Interaction terms were employed to assess heterogeneities among each subgroup, revealing a significant difference specifically in alcohol consumption. This suggests that the positive association between the TyG index and the likelihood of CVD is dependent on the drinking status of the participants.

**Conclusion:**

A higher TyG index is linked to an increased likelihood of CVD in US adults aged ≥ 60 years. TyG index is anticipated to emerge as a more effective metric for identifying populations at early likelihood of CVD.

**Supplementary Information:**

The online version contains supplementary material available at 10.1186/s12933-024-02248-5.

## Introduction

Cardiovascular disease (CVD) remains a major cause of human mortality, posing a threat to a healthy lifespan and contributing to escalating healthcare costs, as well as a critical public health issue of global concern [1]. The incidence and mortality of CVD have been on the rise. The reported number of people with CVD increased from 271 million in 1990 to 523 million in 2019, and the number of deaths due to CVD escalated from 12.1 million in 1990 to 18.6 million in 2019 [2]. Given the escalating incidence and mortality rates of CVD, it is crucial to delve into the factors influencing CVD and identify preventive strategies to delay and mitigate the likelihood of CVD.

Insulin resistance (IR) is a state characterized by decreased sensitivity and response of the body to insulin, manifested by a reduced rate of glucose uptake by the tissues and inhibition of hepatic glucose production, ultimately leading to hyperglycemia [3]. Insulin resistance is considered a precursor phenomenon to type 2 diabetes. Assessment methods for IR such as the hyperinsulin- normoglycemic clamp technique and homeostasis model assessment of IR (HOMA-IR) based on fasting blood glucose and insulin levels have limited their use in large-scale clinical studies due to their high price [4, 5]. The triglyceride-glucose (TyG) index is calculated as the product of the logarithm of fasting triglycerides and fasting glucose. It has gained recognition as a new and reliable indicator of IR due to its advantages of being cost-effective, convenient, and easily obtainable [6]. In comparison to HOMA-IR (AUC: 0.513), the TyG index (AUC: 0.640) demonstrated superior predictability of type 2 diabetes prevalence [7]. The TyG index has been identified as associated with poor prognosis in diseases such as atherosclerosis, coronary artery stenosis, and acute heart failure [8–10]. Some studies have reported higher TyG index levels in younger patients[11]. Notably, in CVD patients under 65 years of age, elevated TyG levels were significantly associated with higher CVD mortality [12]. However, the association of age with the TyG index and cardiovascular likelihood remains highly controversial, and there is a scarcity of large-scale studies investigating the association between the TyG index and CVD likelihood in the older population in the U.S. The objective of our study was to determine whether the TyG index holds prognostic value for CVD likelihood in the older population of the U.S.

## Materials and methods

### Study design

The National Health and Nutrition Examination Survey (NHANES) is a pivotal research program dedicated to assessing the health and nutritional status of both adults and children residing in the United States. The Centers for Disease Control and Prevention (CDC) is tasked with providing health statistics for the nation, and NHANES protocols have received approval from the Research Ethics Review Board of NCHS. To protect participants’ rights, NHANES has obtained informed written consent from all individuals involved in the study. Furthermore, the datasets generated and analyzed in this study are readily accessible on the official NHANES website at https://www.cdc.gov/nchs/nhanes/index.html.

### Study population

**Fig. 1:**
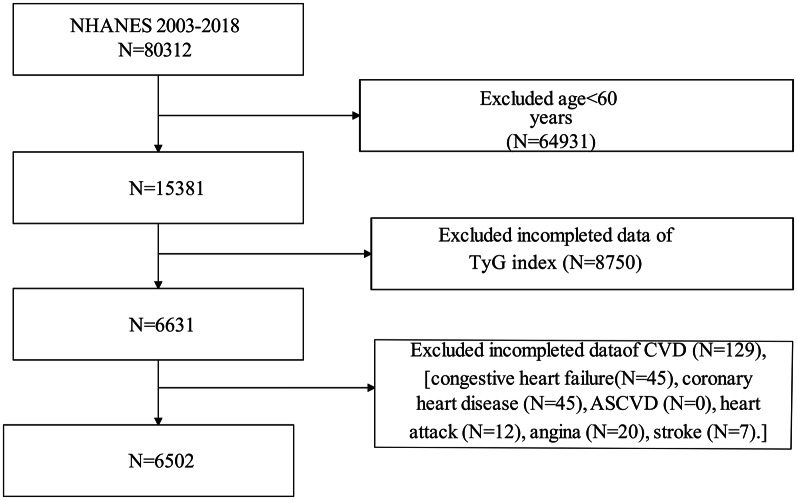
Flowchart of the sample selection from National Health and Nutrition Examination Survey (NHANES) 2003–2018

We utilized continuous NHANES data from survey cycles 2003–2018, collected at 2-year intervals, for our initial sample. These survey cycles provided comprehensive information on the TyG index and various cardiovascular conditions, including congestive heart failure (CHF), coronary heart disease (CHD), atherosclerotic cardiovascular disease (ASCVD), heart attack, angina, and stroke. Initially, our cross-sectional study enrolled 80,312 participants. Following exclusions for individuals under 60 years of age (N = 64,931) and those with missing data on the TyG index (N = 8750) and specific cardiovascular conditions (N [CVD] = 129 [CHF: 45; CHD: 45; ASCVD: 0; heart attack: 12; angina: 20; stroke:7]), the final analysis included 6502 participants (Fig. 1).

### Assessment of TyG index

TyG was chosen as the exposure variable, and we computed the TyG index using the formulation: Ln [triglycerides (mg/dl) × fasting glucose (mg/dl)/2] [13, 14]. The concentrations of triglycerides and fasting glucose were determined through an enzymatic assay conducted on an automatic biochemistry analyzer. Serum triglyceride concentration was measured utilizing the Roche Modular P and Roche Cobas 6000 chemistry analyzers. Fasting plasma glucose levels were assessed through the hexokinase-mediated reaction using the Roche/Hitachi Cobas C 501 chemistry analyzer.

### Assessment of CVD

The medical conditions section, identified by the variable name prefix MCQ, encompasses self- and proxy-reported personal interview data covering an extensive range of health conditions and medical history for both children and adults. This section includes inquiries such as ‘Has a doctor or other health professional ever informed you/SP that you/he/she… had congestive heart failure, coronary heart disease, angina (also called angina pectoris), heart attack (also called myocardial infarction), stroke, etc.?’ These questions, labeled as MCQ160B-F in the household questionnaires administered during home interviews, were utilized to identify participants with a history of CVD if they responded ‘yes’ to any of these questions. We defined a composite endpoint for CVD, comprising CHD, ASCVD, CHF, heart attack, angina, and stroke. Additionally, we separately analyzed events related to CHD, ASCVD, CHF, angina, stroke, and heart attack as secondary outcomes.

### Assessment of covariates

Information on various demographic and health-related factors was collected, including age, gender, race, education level, the ratio of family income to poverty (PIR), body mass index (BMI), serum creatinine, serum uric acid, total cholesterol, HbA1c, albumin/creatinine ratio (ACR), estimated glomerular filtration rate (eGFR), systolic blood pressure, diastolic blood pressure, diabetes (DM), hypertension, smoking, and alcohol consumption status. Fasting venous blood was collected and measured in the laboratory. For laboratory data requiring blood collection, the NHANES study was designed to be estimated only in subjects 12 years of age and older who had fasted for at least 8.5 h or more, but less than 24 h, in the morning. DM was defined as either treatment or medical diagnosis of hyperglycemia with hemoglobin A1c ≥ 6.5%, fasting blood glucose ≥ 126 mg/dL, or a 2-h blood glucose ≥ 200 mg/dL [15]. Hypertension was defined as the use of antihypertensive medications, a medical diagnosis of hypertension, or three consecutive measurements of systolic blood pressure ≥ 140 mmHg or diastolic blood pressure ≥ 90 mmHg [16]. BMI was computed by dividing weight (in kilograms) by the square of height (in meters). Participants were classified as normal weight (< 25 kg/m^2^), overweight (25–29.9 kg/m^2^), or obese (≥ 30 kg/m^2^) based on their BMI.

### Statistical analysis

Statistical analyses were performed in accordance with the guidelines established by the CDC. Given the complex probability sample design and oversampling of specific populations in NHANES to ensure representativeness, sample weights were applied to combine data from multiple survey cycles. Study participants were classified into four groups according to quartiles (Q1–Q4) of the TyG index. Continuous variables were reported as means ± standard error, and categorical variables were presented as percentages. The weighted one-way ANOVA was utilized for continuous variables, and the weighted chi-square test was employed for categorical variables to assess differences in the descriptive analyses. Multivariable logistic regression was employed to investigate the correlation between the TyG index and the likelihood of CVD using three distinct models for statistical inference. Model 1 was unadjusted, Model 2 was adjusted for age, gender, and race, and Model 3 was adjusted for age, gender, race, education level, PIR, BMI, serum creatinine, serum uric acid, total cholesterol, HbA1c, ACR, eGFR, systolic blood pressure, diastolic blood pressure, DM, hypertension, smoking, and alcohol consumption status. In sensitivity analyses, the TyG index was categorized into quartiles to assess the robustness of the results, and the likelihood of CVD across these quartiles was evaluated. Additionally, restricted cubic spline (RCS) analysis with three piecewise points was employed to explore potential nonlinear relationships between the TyG index and the likelihood of CVD. For subgroup analysis of the association between the TyG index and the likelihood of CVD, the data were stratified by gender (male/female), BMI (normal weight/overweight/obesity), hypertension (yes/no), DM (yes/no), smoking status (never/former/now) and alcohol use (yes/no). These stratified factors were also considered as potential effect modifiers [13, 17]. A significance level of two-sided P < 0.05 was used to indicate statistical significance. All analyses were conducted using R version 4.3.2 (http://www.R-project.org, The R Foundation).

## Results

### Baseline characteristics of study participants

Table 1 shows the baseline characteristics of the study participants stratified by quartile of the TyG index. A total of 6502 participants were included in our study, with an average age of 69.80 ± 0.14 years, and 54.68% of them were female, 45.32% were male. The average TyG index in the recruited subjects was 8.75 ± 0.01, and the range of TyG index for quartiles 1–4 were 6.72–8.34, 8.34–8.73, 8.73–9.15, 9.15–12.55, respectively. The average prevalence of CVD was 24.31% overall. Participants in the higher TyG quartiles showed high rates of CVD (Quartile 1: 19.91%; Quartile 2: 21.65%; Quartile 3: 23.82%; Quartile 4: 32.43%), CHD (Quartile 1: 8.57%; Quartile 2: 9.63%; Quartile 3: 10.17%; Quartile 4: 14.57%), CHF (Quartile 1: 5.28%; Quartile 2: 5.87%; Quartile 3: 6.56%; Quartile 4: 10.47%), ASCVD (Quartile 1: 18.20%; Quartile 2: 20.24%; Quartile 3: 22.01%; Quartile 4: 29.37%), angina (Quartile 1: 3.98%; Quartile 2: 5.32%; Quartile 3: 6.14%; Quartile 4: 9.45%), and heart attack (Quartile 1: 7.52%; Quartile 2: 9.32%; Quartile 3: 10.05%; Quartile 4: 12.29%). Participants with a higher TyG index were found to be more likely to be Mexican American, Non-Hispanic White, and obese when compared to participants in the lowest quartile. Moreover, notable differences in biochemical indicators were observed between the groups, with participants in the highest quartile exhibiting significantly high levels of serum creatinine, serum uric acid, total cholesterol, triglyceride, fast glucose, HbA1c, and ACR in comparison to those in the lowest quartile. Furthermore, participants in the highest quartile also had a higher rate of DM and hypertension compared to the lowest quartile.

**Table 1:** Weighted baseline characteristics of the study population

TyG index	All participants	Quartile 1 (6.72–8.34)	Quartile 2 (8.34–8.73)	Quartile 3 (8.73–9.15)	Quartile 4 (9.15–12.55)	P value
Age (year)	69.80 (0.24)	69.63 (0.22)	70.07 (0.27)	70.09 (0.21)	69.42 (0.22)	0.05
Serum creatinine (mg/dl)	0.98 (0.01)	0.95 (0.01)	0.97 (0.01)	0.98 (0.02)	1.02 (0.02)	**0.01**
Serum uric acid (umol/L)	339.92 (1.58)	315.41 (2.69)	331.66 (2.57)	348.60 (2.76)	356.94 (3.94)	** < 0.0001**
Total cholesterol (mg/dl)	194.30 (0.82)	185.78 (1.42)	194.49 (1.39)	195.73 (1.64)	201.95 (1.62)	** < 0.0001**
LDL-C (mg/dl)	111.76 (0.71)	104.55 (1.16)	112.54 (1.38)	114.24 (1.24)	116.23 (1.44)	** < 0.0001**
Triglyceride (mg/dl)	130.66 (1.69)	63.75 (0.57)	99.09 (0.57)	137.44 (0.93)	230.07 (3.46)	** < 0.0001**
Fast glucose (mg/dl)	114.88 (0.60)	100.43 (0.54)	106.08 (0.70)	114.25 (0.77)	140.63 (1.75)	** < 0.0001**
HbA1c (%)	5.94 (0.02)	5.60 (0.02)	5.75 (0.02)	5.95 (0.03)	6.52 (0.06)	** < 0.0001**
BMI (Kg/m^2^)	29.04 (0.13)	26.86 (0.22)	28.23 (0.21)	30.01 (0.22)	31.26 (0.24)	** < 0.0001**
ACR (mg/g)	56.41 (4.50)	33.80 (5.19)	44.85 (5.05)	51.81 (10.23)	98.13 (12.50)	** < 0.001**
eGFR (mL/min/1.73 m^2^)	73.63 (0.30)	75.68 (0.62)	73.66 (0.66)	72.79 (0.55)	72.25 (0.64)	**0.001**
Systolic blood pressure (mmHg)	130.93 (0.38)	128.06 (0.74)	130.82 (0.63)	131.56 (0.71)	133.58 (0.68)	** < 0.0001**
Diastolic blood pressure (mmHg)	66.92 (0.28)	66.46 (0.43)	67.17 (0.50)	66.59 (0.47)	67.50 (0.44)	0.20
TyG index	8.75 (0.12)	8.03 (0.11)	8.54 (0.12)	8.93 (0.11)	9.57 (0.12)	** < 0.0001**
Gender, % (SE)						0.81
Female	54.68 (1.76)	55.48 (1.71)	55.29 (1.53)	54.62 (1.67)	53.24 (1.81)	
Male	45.32 (1.76)	44.52 (1.71)	44.71 (1.53)	45.38 (1.67)	46.76 (1.81)	
Races, % (SE)						** < 0.0001**
Mexican American	3.96 (0.43)	2.18 (0.36)	3.99 (0.53)	4.71 (0.60)	5.09 (0.68)	
Non-Hispanic Black	8.08 (0.85)	12.75 (0.99)	8.40 (0.74)	5.67 (0.54)	5.15 (0.56)	
Non-Hispanic White	78.92 (1.52)	77.70 (1.55)	78.22 (1.50)	79.73 (1.30)	80.13 (1.39)	
Others	9.05 (0.94)	7.38 (0.92)	9.39 (0.98)	9.90 (0.92)	9.63 (0.97)	
Educational levels, % (SE)						** < 0.0001**
Less than 9th grade	9.19 (0.89)	6.67 (0.62)	9.30 (0.84)	9.47 (0.94)	11.56 (0.92)	
9-11th grade	10.93 (1.13)	10.15 (0.95)	10.06 (0.95)	11.71 (1.13)	11.89 (1.13)	
High school graduate	25.52 (1.72)	22.77 (1.64)	24.37 (1.51)	26.30 (1.55)	28.93 (1.68)	
Some college or AA degree	27.64 (1.71)	25.81 (1.67)	29.94 (1.65)	26.62 (2.00)	28.32 (1.75)	
College graduate or above	26.71 (1.95)	34.61 (2.03)	26.32 (1.86)	25.89 (2.04)	19.31 (1.45)	
PIR,% (SE)						** < 0.0001**
< 1	8.58 (0.88)	8.36 (0.82)	8.82 (0.89)	10.57 (1.03)	9.83 (0.80)	
1–4	50.86 (1.92)	49.73 (2.40)	55.69 (1.83)	57.18 (1.99)	60.27 (1.88)	
> 4	32.01 (2.18)	41.91 (2.58)	35.49 (1.90)	32.25 (2.16)	29.90 (1.91)	
BMI, % (SE)						** < 0.0001**
Normal weight	26.13 (1.87)	42.19 (2.06)	29.67 (1.81)	19.89 (1.40)	12.87 (1.08)	
Overweight	35.47 (1.92)	36.12 (1.85)	38.85 (1.93)	35.94 (1.74)	32.97 (1.47)	
Obesity	36.89 (1.52)	21.69 (1.42)	31.47 (1.53)	44.17 (1.85)	54.17 (1.57)	
Smoke, % (SE)						0.05
Never	48.28 (1.95)	49.32 (2.02)	50.43 (1.85)	50.20 (1.62)	43.05 (1.78)	
Former	40.45 (1.84)	40.83 (1.87)	38.99 (1.78)	37.08 (1.48)	45.25 (1.77)	
Now	11.18 (1.24)	9.84 (1.00)	10.59 (1.18)	12.72 (1.08)	11.70 (1.36)	
Alcohol use, % (SE)	70.24 (1.55)	72.53 (1.48)	72.45 (1.52)	70.63 (1.51)	65.01 (1.90)	**0.003**
Hypertension, % (SE)	66.81 (1.94)	56.44 (1.96)	64.38 (1.80)	69.40 (1.68)	78.05 (1.65)	** < 0.0001**
DM, % (SE)	31.68 (1.74)	13.59 (1.17)	20.69 (1.38)	35.29 (1.83)	59.29 (1.91)	** < 0.0001**
CVD, % (SE)	24.31 (1.38)	19.91 (1.32)	21.65 (1.40)	23.82 (1.40)	32.43 (1.79)	** < 0.0001**
Stroke, % (SE)	8.00 (0.68)	7.30 (0.68)	7.17 (0.91)	7.43 (0.74)	10.22(0.94)	0.06
CHD, % (SE)	10.67 (0.98)	8.57 (0.96)	9.63 (0.85)	10.17 (0.99)	14.57 (1.49)	** < 0.001**
CHF, % (SE)	6.99 (0.66)	5.28 (0.67)	5.87 (0.66)	6.56 (0.83)	10.47 (0.98)	** < 0.0001**
ASCVD, % (SE)	22.33 (1.31)	18.20 (1.28)	20.24 (1.35)	22.01 (1.41)	29.37 (1.77)	** < 0.0001**
Heart attack, % (SE)	9.74 (0.96)	7.52 (0.97)	9.32 (0.83)	10.05 (0.98)	12.29 (1.23)	**0.01**
Angina, % (SE)	6.16 (0.74)	3.98 (0.57)	5.32 (0.69)	6.14 (0.83)	9.45 (1.29)	** < 0.001**

Bold indicates statistical significance

The weighted one-way ANOVA was utilized for continuous variables, and the weighted chi-square test was employed for categorical variables to assess differences in the descriptive analyses

*LDL-C* low-density lipoprotein cholesterol, *HDL-C* high-density lipoprotein cholesterol, *ACR* urinary albumin: creatinine ratio, *eGFR* estimated-glomerular filtration rate, *BMI* body mass index, *PIR* family income-poverty ratio, *DM* diabetes, *CHF* congestive heart failure, *CVD* cardiovascular disease, *CHD* congestive heart disease, *ASCVD* atherosclerotic cardiovascular disease

Our discoveries unveiled notable distinctions between male and female participants. In contrast to their female counterparts, male participants exhibited elevated levels of serum uric acid and fasting glucose, increased TyG index, elevated prevalence of obesity and diabetes mellitus, and a higher occurrence of CVD, CHD, CHF, ASCVD, heart attack, and angina (Additional file 1: Table S1).

### Relationship of TyG index with the likelihood of CVD

For CVD, a possible association between the TyG index and the likelihood of CVD was observed (Table 2). In the fully adjusted model (Model 3), this positive association remained stable (OR = 1.30, 95%CI: 1.09–1,55, p = 0.03). Additionally, we conducted sensitivity analysis by converting the TyG index from a continuous variable to a categorical variable (quartiles). The multivariable-adjusted OR and 95%CI from the lowest to the highest TyG index quartile were 1.00 (reference), 1.12 (1.03–1.20), 1.21 (1.04–1.37), and 1.51 (1.14–1.99), respectively.

**Table 2:** The association between TyG index and the risk of CVD

CVD	OR (95%CI)		
	Model 1	Model 2	Model 3
TyG index (continuous)	1.50 (1.31, 1.72), **p < 0.0001**	1.58 (1.36, 1.82), **p < 0.0001**	1.30 (1.09, 1.55) **p = 0.003**
TyG index (quartiles)			
Quartile 1	Reference	Reference	Reference
Quartile 2	1.21 (1.03, 1.39), **p = 0.03**	1.24 (1.09, 1.40), **p = 0.03**	1.12 (1.03, 1.20), **p = 0.03**
Quartile 3	1.26 (1.01, 1.57), **p = 0.04**	1.28 (1.02, 1.61), **p = 0.03**	1.21 (1.04, 1.37), **p = 0.03**
Quartile 4	1.93 (1.53, 2,43) **p < 0.0001**	2.08 (1.65, 2.64), **p < 0.0001**	1.51 (1.14, 1.99), **p = 0.005**

Bold indicates statistical significance

Model 1: No covariates were adjusted

Model 2: Age, gender, and race were adjusted

Model 3: Age, gender, race, education level, PIR, BMI, serum creatinine, serum uric acid, total cholesterol, HbA1c, ACR, eGFR, systolic blood pressure, diastolic blood pressure, DM, hypertension, smoking and alcohol consumption status were adjusted

*OR* odds ratio, *95%CI* 95% Confidence Interval, *CVD* cardiovascular disease

No significant association between the TyG index and the odds of stroke and CHF was observed in our study (Table 3 and Table 4).

**Table 3:** The association between TyG index and the risk of stroke

Stroke	OR (95%CI)		
	Model 1	Model 2	Model 3
TyG index (continuous)	1.26 (1.06, 1.50), **p = 0.01**	1.33 (1.11, 1.61), **p = 0.002**	1.04 (0.78, 1.40) p = 0.77
TyG index (quartiles)			
Quartile 1	Reference	Reference	Reference
Quartile 2	1.05 (0.75, 1.37), p = 0.91	1.04 (0.70, 1.38), p = 0.91	1.02 (0.75, 1.28), p = 0.29
Quartile 3	1.08 (0.78, 1.38), p = 0.89	1.09 (0.79, 1.38), p = 0.76	1.10 (0.81, 1.39), p = 0.28
Quartile 4	1.44 (1.09, 1.92) **p = 0.01**	1.56 (1.16, 2.09), **p = 0.004**	1.29 (0.87, 1.70), p = 0.47

Bold indicates statistical significance

Model 1: No covariates were adjusted

Model 2: Age, gender, and race were adjusted

Model 3: Age, gender, race, education level, PIR, BMI, serum creatinine, serum uric acid, total cholesterol, HbA1c, ACR, eGFR, systolic blood pressure, diastolic blood pressure, DM, hypertension, smoking and alcohol consumption status were adjusted

*OR* odds ratio, *95%CI* 95% Confidence Interval

**Table 4:** The association between TyG index and the risk of CHF

CHF	OR (95%CI)		
	Model 1	Model 2	Model 3
TyG index (continuous)	1.53 (1.26, 1.86), **p < 0.0001**	1.63 (1.33, 1.99), **p < 0.0001**	1.18 (0.90, 1.54) p = 0.22
TyG index (quartiles)			
Quartile 1	Reference	Reference	Reference
Quartile 2	1.12 (0.80, 1.56), p = 0.50	1.13 (0.81, 1.56), p = 0.47	1.09 (0.85, 1.32), p = 0.60
Quartile 3	1.26 (0.85, 1.86), p = 0.89	1.30 (0.79, 1.38), p = 0.76	1.18 (0.89, 1.47), p = 0.75
Quartile 4	2.10 (1.45, 3.04) **P < 0.001**	2.27 (1.56, 3.31), **P < 0.0001**	1.95 (0.87, 3.02), p = 0.19

Bold indicates statistical significance

Model 1: No covariates were adjusted

Model 2: Age, gender, and race were adjusted

Model 3: Age, gender, race, education level, PIR, BMI, serum creatinine, serum uric acid, total cholesterol, HbA1c, ACR, eGFR, systolic blood pressure, diastolic blood pressure, DM, hypertension, smoking and alcohol consumption status were adjusted

*OR* odds ratio, *95%CI* 95% Confidence Interval, *CHF* congestive heart failure

Concerning the likelihood of CHD and heart attack (Table 5 and Table 6), we observed that an increased TyG index was associated with higher odds of CHD (Model 3: OR = 1.40, 95%CI: 1.11–1.78, p = 0.01) and heart attack (Model 3: OR = 1.40, 95%CI: 1.11–1.78, p = 0.01). In the sensitivity analysis, the adjusted OR for the quartile (reference to Quartile 1) was 1.61 (95%CI: 1.10–2.37) and 1.87 (95%CI: 1.21–2.53) for CHD and heart attack, respectively, suggesting a stable positive relationship between an elevated TyG index and increased likelihood of CHD and heart attack with statistical significance.

**Table 5:** The association between TyG index and the risk of CHD

CHD	OR (95%CI)		
	Model 1	Model 2	Model 3
TyG index (continuous)	1.48 (1.22, 1.78), **p < 0.0001**	1.47 (1.21, 1.79), **p < 0.001**	1.40 (1.11, 1.78) **p = 0.01**
TyG index (quartiles)			
Quartile 1	Reference	Reference	Reference
Quartile 2	1.21 (0.89, 1.63), p = 0.21	1.18 (0.87, 1.61), p = 0.29	1.20 (0.85, 1.70), p = 0.31
Quartile 3	1.40 (0.94, 1.55), p = 0.41	1.20 (0.89, 1.51), p = 0.57	1.34 (0.93, 1.74), p = 0.59
Quartile 4	1.82 (1.30, 2.54) **P < 0.001**	1.81 (1.31, 2.52), **P < 0.001**	1.61 (1.10, 2.37), **p = 0.02**

Bold indicates statistical significance

*OR* odds ratio, *95%CI* 95% Confidence Interval, *CHD*congestive heart disease

Model 1: No covariates were adjusted

Model 2: Age, gender, and race were adjusted

Model 3: Age, gender, race, education level, PIR, BMI, serum creatinine, serum uric acid, total cholesterol, HbA1c, ACR, eGFR, systolic blood pressure, diastolic blood pressure, DM, hypertension, smoking and alcohol consumption status were adjusted

**Table 6:** The association between TyG index and the risk of heart attack

Heart attack	OR (95%CI)		
	Model 1	Model 2	Model 3
TyG index (continuous)	1.35 (1.13, 1.62), **p = 0.001**	1.36 (1.13, 1.64), **P = 0.001**	1.32 (1.16, 1.47) **p = 0.001**
TyG index (quartiles)			
Quartile 1	Reference	Reference	Reference
Quartile 2	1.38 (0.99, 1.92), p = 0.06	1.46 (0.98, 1.95), p = 0.07	1.36 (0.86, 1.85), p = 0.31
Quartile 3	1.43 (0.91, 1.96), p = 0.16	1.53 (0.89, 2.17), p = 0.18	1.43 (0.91, 1.94), p = 0.59
Quartile 4	1.72 (1.02, 2.48) **p = 0.004**	1.76 (1.22, 2.55), **P = 0.003**	1.87 (1.21, 2.53), **p = 0.003**

Bold indicates statistical significance

Model 1: No covariates were adjusted

Model 2: Age, gender, and race were adjusted

Model 3: Age, gender, race, education level, PIR, BMI, serum creatinine, serum uric acid, total cholesterol, HbA1c, ACR, eGFR, systolic blood pressure, diastolic blood pressure, DM, hypertension, smoking and alcohol consumption status were adjusted

*OR* odds ratio, *95%CI* 95% Confidence Interval

Table 7 and Table 8 shows a significant increase between the TyG index and the likelihood of angina (Model 3: OR: 1.39, 95%CI: 1.04–1.87, p = 0.03) and ASCVD (Model 3: OR: 1.26, 95%CI: 1.04–1.52, p = 0.02). In the sensitivity analyses, in fully adjusted Model 3, the highest quartile of the TyG index demonstrated an increase in the likelihood of both angina (OR: 1.65, 95%CI: 1.04–2.62, p < 0.0001) and ASCVD (OR: 1.44, 95%CI: 1.07–1.92, p < 0.0001).

**Table 7:** The association between TyG index and the risk of angina

Angina	OR (95%CI)		
	Model 1	Model 2	Model 3
TyG index (continuous)	1.70 (1.36, 2.11), **p < 0.0001**	1.70 (1.36, 2.12), **p < 0.0001**	1.39 (1.04, 1.87) **p = 0.03**
TyG index (quartiles)			
Quartile 1	Reference	Reference	Reference
Quartile 2	1.36 (1.01, 2.04), **p = 0.04**	1.51 (1.02, 2.01), **p = 0.01**	1.48 (1.05, 1.90), **p = 0.03**
Quartile 3	1.58 (1.04, 2.42), **p = 0.03**	1.55 (1.01, 2.37), **p = 0.04**	1.50 (1.07, 1.92), **p = 0.04**
Quartile 4	2.52 (1.72, 3.68) **p < 0.0001**	2.51 (1.72, 3.67), **p < 0.0001**	1.65 (1.04, 2.62), **p < 0.0001**

Bold indicates statistical significance

Model 1: No covariates were adjusted

Model 2: Age, gender, and race were adjusted

Model 3: Age, gender, race, education level, PIR, BMI, serum creatinine, serum uric acid, total cholesterol, HbA1c, ACR, eGFR, systolic blood pressure, diastolic blood pressure, DM, hypertension, smoking and alcohol consumption status were adjusted

*OR* odds ratio, *95%CI* 95% Confidence Interval

**Table 8:** The association between TyG index and the risk of ASCVD

ASCVD	OR (95%CI)		
	Model 1	Model 2	Model 3
TyG index (continuous)	1.46 (1.27, 1.69), **p < 0.0001**	1.52 (1.31, 1.76), **p < 0.0001**	1.26 (1.04, 1.52) **p = 0.02**
TyG index (quartiles)			
Quartile 1	Reference	Reference	Reference
Quartile 2	1.22 (1.01, 1.43), **p = 0.04**	1.24 (1.03, 1.44), **p = 0.02**	1.21 (1.08, 1.33), **p = 0.03**
Quartile 3	1.27 (1.04, 1.58), **p = 0.04**	1.28 (1.02, 1.61), **p = 0.04**	1.25 (1.11, 1.39), **p = 0.04**
Quartile 4	1.87 (1.48, 2.36) **p < 0.0001**	1.98 (1.56, 2.51), **p < 0.0001**	1.44 (1.07, 1.92), **p < 0.0001**

Bold indicates statistical significance

Model 1: No covariates were adjusted

Model 2: Age, gender, and race were adjusted

Model 3: Age, gender, race, education level, PIR, BMI, serum creatinine, serum uric acid, total cholesterol, HbA1c, ACR, eGFR, systolic blood pressure, diastolic blood pressure, DM, hypertension, smoking and alcohol consumption status were adjusted

*OR* odds ratio, *95%CI* 95% Confidence Interval, *ASCVD* atherosclerotic cardiovascular disease

### RCS analysis

**Fig. 2:**
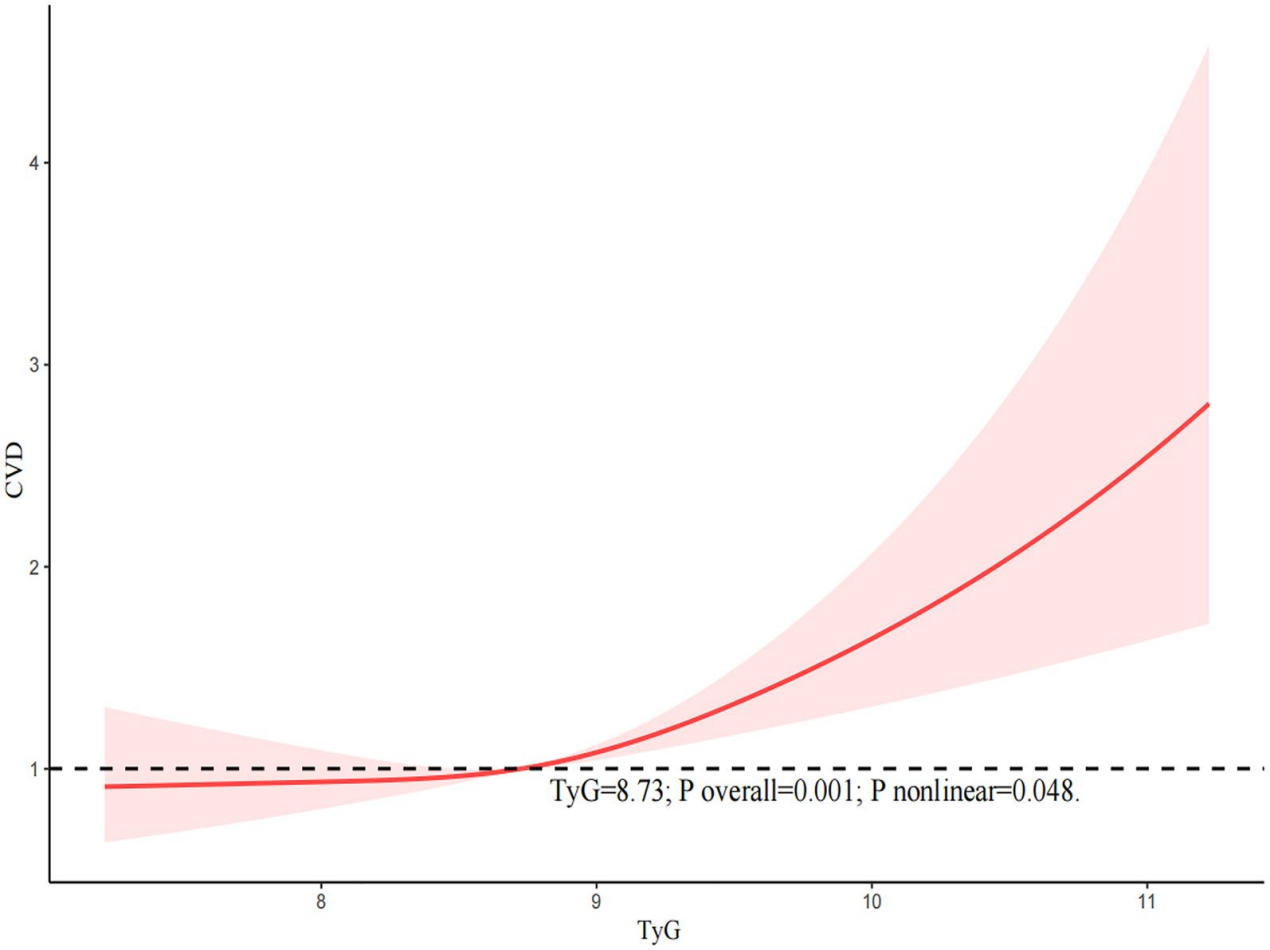
The restricted cubic spline (RCS) analysis between the TyG index and the risk of cardiovascular disease (CVD)

We utilized RCS curves to examine the potential nonlinearity in the relationship between the TyG index and the likelihood of CVD, as illstrates in Fig. 2. Our findings suggested a nonlinear association (P nonlinear = 0.048). Below a threshold of 8.73 of the TyG index, we observed a gently increasing relationship between the TyG index and CVD likelihood, and to the right of the inflection point of 8.73, we observed a significantly steeper upward trend in the relationship between the TyG index and CVD.

**Fig. 3:**
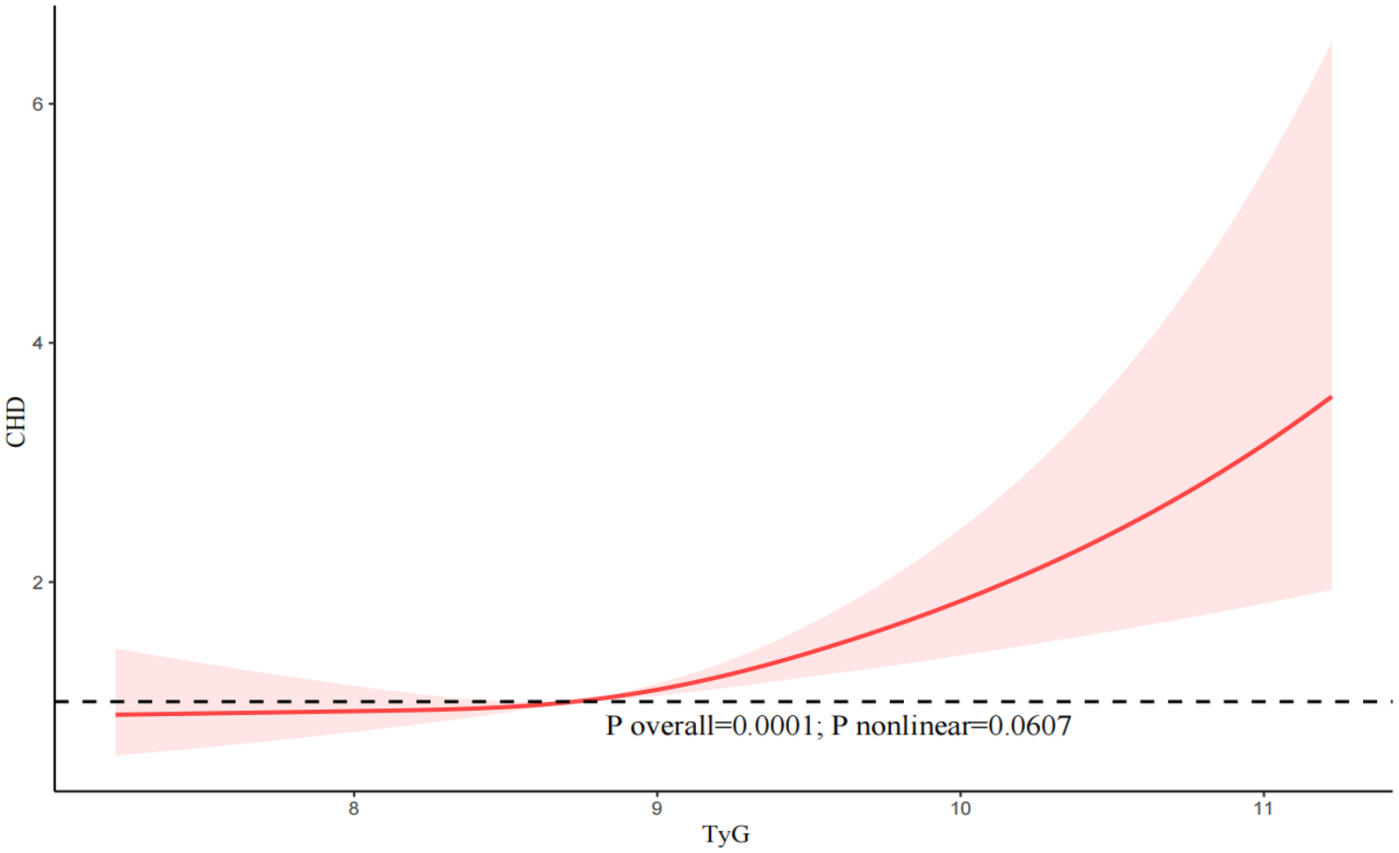
The restricted cubic spline (RCS) analysis between the TyG index and the risk of congestive heart disease (CHD)

**Fig. 4:**
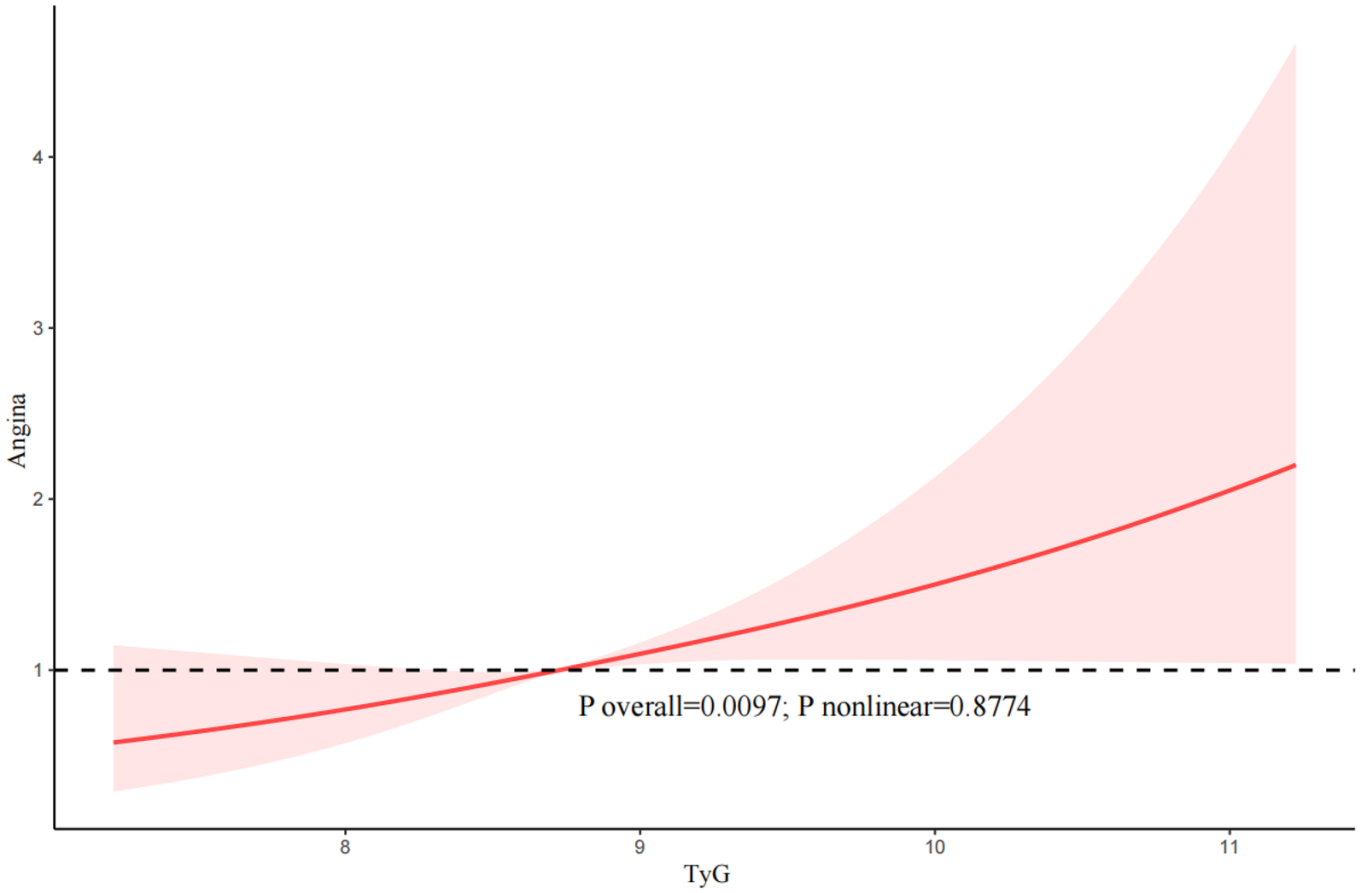
The restricted cubic spline (RCS) analysis between the TyG index and the risk of angina

**Fig. 5:**
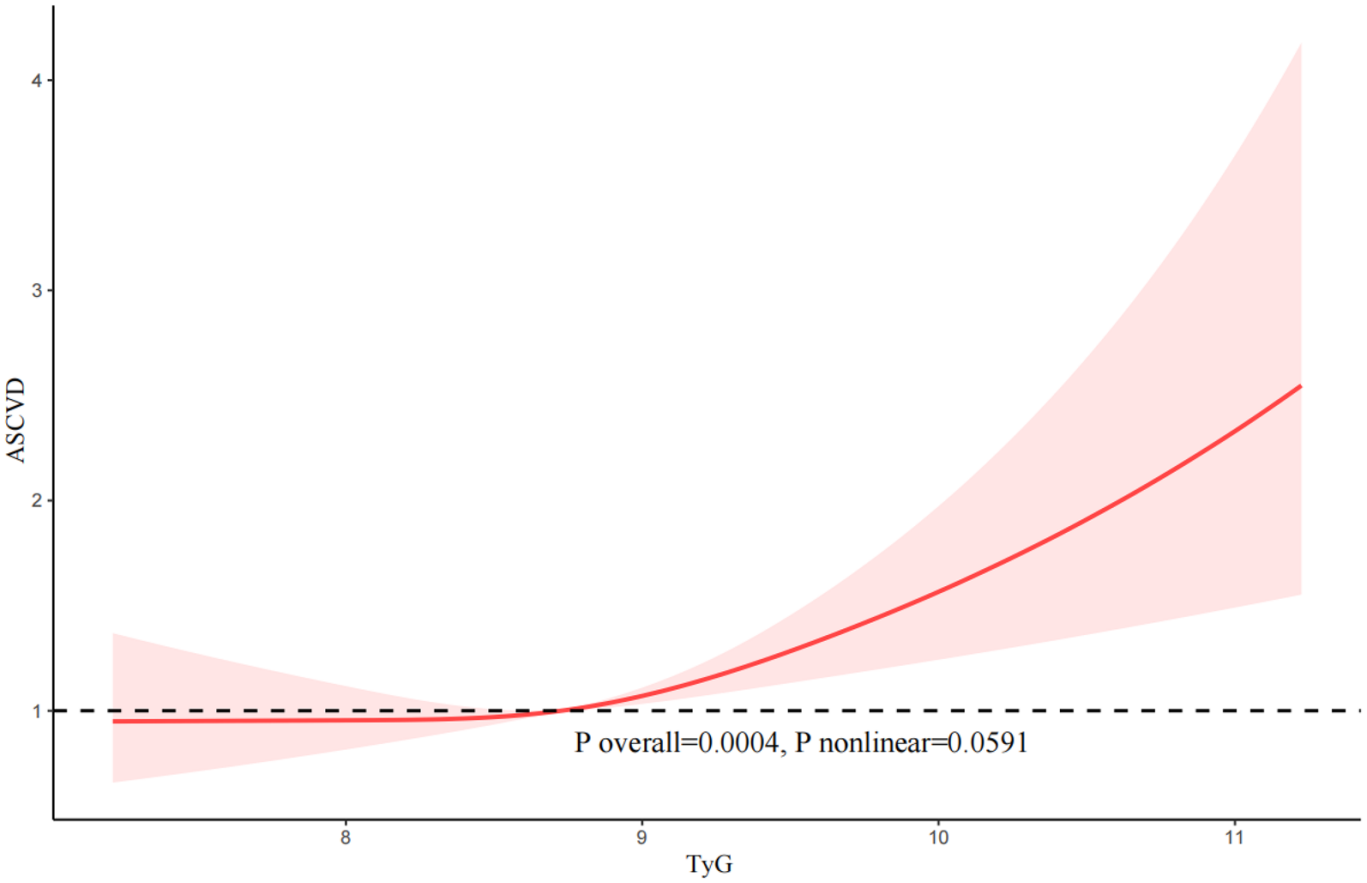
The restricted cubic spline (RCS) analysis between the TyG index and the risk of atherosclerotic cardiovascular disease (ASCVD)

In addition, our results indicated that there was an approximately linear relationship between the TyG index and the likelihood of CHD (P nonlinear = 0.0607), angina (P nonlinear = 0.8774), and ASCVD (P nonlinear = 0.0591) (Fig. 3, 4, 5).

### Subgroup analysis

We conducted subgroup analyses and interaction tests to explore the consistency of the relationship between the TyG index and the likelihood of CVD, CHD, ASCVD, angina, and heart attack across various population subgroups. These subgroups included gender, BMI, hypertension, DM, smoking status, and alcohol consumption.

**Fig. 6:**
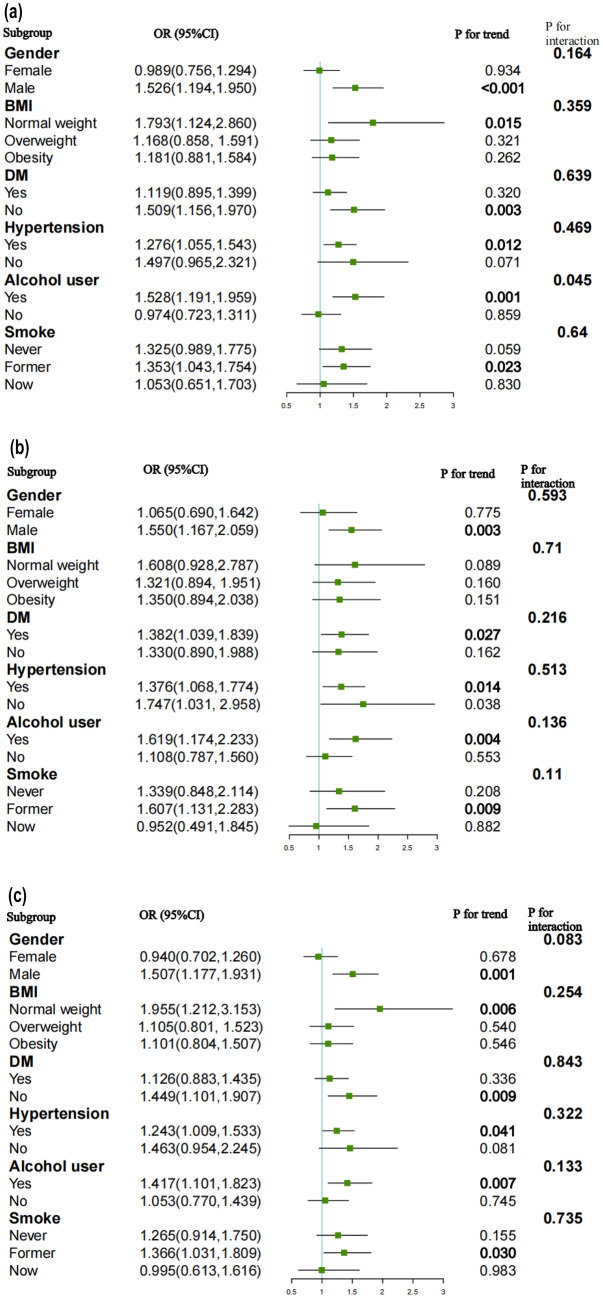

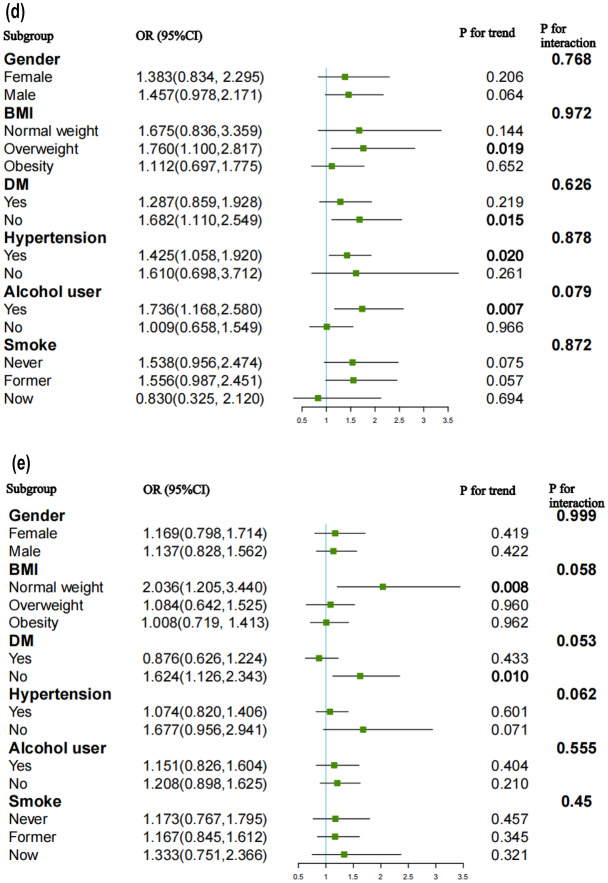
Subgroup analysis for the association between the TyG index and the risk of CVD, CHD, ASCVD, angina and heart attack. **a** Subgroup analysis for the association between the TyG index and the risk of CVD. **b** Subgroup analysis for the association between the TyG index and the risk of CHD. **c** Subgroup analysis for the association between the TyG index and the risk of ASCVD. **d** Subgroup analysis for the association between the TyG index and the risk of angina. **e** Subgroup analysis for the association between the TyG index and the risk of heart attack

The likelihood of CVD increased in participants who were male (OR = 1.526, 95%CI: 1.194–1.950, p < 0.001), normal weight (OR = 1.793, 95%CI: 1.124–2.860, p = 0.015), former smokers (OR = 1.353, 95%CI: 1.043–1.754, p = 0.023) and alcohol users (OR = 1.528, 95%CI: 1.191–1.959, p = 0.001). Individuals with hypertension (OR = 1.276, 95%CI: 1.055–1.543, p = 0.012) and without DM (OR = 1.509, 95%CI: 1.156–1.970, p = 0.003) also had an elevated likelihood of CVD (Fig. 6a).

Interaction terms were employed to assess heterogeneities among each subgroup, revealing a significant difference specifically in alcohol consumption (P for interaction = 0.045). This indicated that the positive association between the TyG index and the likelihood of CVD was dependent on the drinking status of the participants.

For the likelihood of CHD (Fig. 6b), ASCVD (Fig. 6c), angina (Fig. 6d), and heart attack (Fig. 6e), no statistically significant correlation with the p for interaction was detected, suggesting that there was no dependence on gender, BMI, hypertension, DM, smoking and drinking status for this association. Our results indicated that the positive association between the TyG index and the likelihood of CHD, ASCVD, angina, and heart attack was consistent across populations with different statuses and could be appropriate for various population settings.

### The association of CVD, stroke, CHF, CHD, heart attack, angina and ASCVD on TyG index levels

In addition to this, we further analyzed the changes in the TyG index in individuals with CVD and individuals with different CVD subtypes (Additional file 1: Table S2). In the fully adjusted Model 3, we observed an increase in TyG index of 0.05 and 0.07 units in individuals with CVD (β: 0.05, 95%CI: 0.01–0.09) and CHD (β: 0.07, 95%CI: 0.01–0.13), respectively, compared to participants without CVD and CHD disease. In contrast, no significant change in TyG index increase was observed in participants with stroke, CHF, heart attack, angina, and ASCVD.

We also compared the effect of gender on the change in the TyG index in participants with CVD and CHD (Additional file 1: Table S3). Our results found that the increase in TyG index was more pronounced in men among individuals with CVD (β: 0.116, 95%CI: 0.049–0.182) and CHD (β: 0.129, 95%CI: 0.049–0.208).

Interaction terms were employed to assess heterogeneities among each subgroup, revealing a significant difference specifically in male CVD individuals (P for interaction = 0.047). No statistically significant correlation with the p for interaction was detected in male CHD individuals (P for interaction = 0.286). This indicated that the positive association between the CVD status and TyG index was dependent on the gender of the participants.

## Discussion

In this cross-sectional study that included 6502 adults, we found that a higher TyG index was independently associated with an increased likelihood of of CVD, CHD, angina, ASCVD, and heart attack. The association between the TyG index and the likelihood of CVD was non-linear. Interaction tests indicated that this positive association was dependent on the drinking status of the participants. However, the association between the TyG index and the likelihood of CHD, angina, ASCVD, and heart attack was similar in subgroups stratified by gender, BMI, hypertension, diabetes, smoking, and drinking status. Our study showed that the TyG index could serve as an effective predictor of CVD likelihood in the general population of US adults aged ≥ 60 years. We also observed an increase in TyG index of 0.05 and 0.07 units in individuals with CVD respectively, compared to participants without CVD and CHD. In male individuals with CVD, the increase in the TyG index was more pronounced. This phenomenon may be related to the fact that men are more likely to smoke and drink alcohol and have more risk factors for metabolic diseases [18].

The gold standard for assessing IR, such as the high insulin-normal glucose clamp approach, is less adaptable in a wide range of epidemiologic investigations due to its invasive and costly nature and has been gradually replaced by alternative markers such as HOMA-IR and TyG index [4, 19]. The potential of TyG index as an assessment of IR was suggested by Guerrero et al. in 2010 [20]. Despite HOMA-IR as a common metric used for the assessment of IR, however, the complexity of the technique required for fasting insulin measurements and the high cost of the test have limited its widespread use in the clinical setting [4, 21]. Compared with the HOMA-IR index, the TyG index, which combines fasting glucose and lipid parameters, is easier to obtain, has fewer laboratory operations, is less expensive, and is more amenable to clinical application [22]. Several studies have shown that the TyG index is consistent with or more clearly superior to the HOMA-IR in assessing IR, and it expresses significant sensitivity and specificity [7, 23–25]. In addition to this, the TyG index stands out for its predictive ability for cardiometabolic diseases compared to other alternative markers for assessing IR such as lipid accumulation products and visceral obesity index [26]. In conclusion, TyG index has greater potential for clinical application due to its simplicity of clinical application, utilization of routine laboratory tests, and elimination of the need for insulin measurements.

The correlation between the TyG index and CVD likelihood was reported in previous studies. Wang et al. reported the potential of TyG as a valuable predictor of CHD severity, particularly in pre-diabetic patients [27]. Che et al. identified an elevated TyG index as a predictor for CVD odds in a European population [28]. Liu et al. showed that a higher baseline TyG index was associated with an increased likelihood of future CVD development in postmenopausal women [29]. Moreover, in populations at high.

for CVD, a high TyG index correlated with elevated odds of cardiovascular death, emphasizing the significance of the TyG index in primary CVD prevention [30]. Notably, in non-diabetic populations, individuals with higher TyG index values demonstrated an increased likelihood of impaired cardiovascular health [31].

Chen et al. found that the TyG index showed a significant correlation with cardiovascular disease (CVD) mortality only in people younger than 65 years of age, especially those aged 45–64 years [32]. Liu et al. also found that the TyG index was more predictive of the.

of ischemic stroke death in younger patients, whereas no correlation with the likelihood of ischemic stroke death was observed in older patients [33]. Wang et al. demonstrated that a higher TyG index was not associated with the likelihood of CVD death in older participants, whereas all-cause mortality was higher in patients younger than 65 years of age with a higher TyG index [12]. Ma et al. found a significant association of a higher TyG index with an increased incidence of adverse cardiovascular events in patients younger than 70 years of age, whereas similar results were not observed in patients older than 70 years of age [34]. In a large multicenter study, higher levels of the TyG index were found to increase the likelihood of severe atherosclerotic obstructive coronary heart disease in patients aged 18 to 45 years [35]. In our study, a positive correlation was found between the TyG index and the likelihood of CVD in the older US general population. The exact role of age in mediating the relationship between the TyG index and CVD likelihood still needs to be confirmed by large-scale studies. Measurement of the TyG index in the elderly is susceptible to more confounding factors, such as the presence of various diseases and concomitant poor nutritional status, which may lead to inaccurate measurement of the TyG index.

In our subgroup analysis in this study, a positive correlation between the TyG index and CVD likelihood was found to depend on drinking status. A Korean study discovered that among obese Korean men, both high and moderate alcohol consumption was associated with a higher incidence of impaired fasting glucose or diabetes [36]. Another study involving Korean citizens aged 30 and above found that even occasional high-risk drinking was associated with an increased likelihood of impaired fasting glucose compared to non-risk drinking [37]. In non-obese, non-diabetic Japanese male citizens, even a small amount of alcohol consumption may lead to impaired fasting blood glucose [38]. Li et al. discovered that occasional or moderate alcohol consumption was not associated with the odds of developing hyperglycemia compared to never drinking alcohol [39]. However, heavy alcohol consumption was linked to a higher likelihood of developing hyperglycemia. Park KY et al. found that high-risk drinking (30 g or more of alcohol per day for men and 20 g or more for women) exacerbated the incidence of hypertriglyceridemia [40]. While the risk of low HDL cholesterol decreased with increasing frequency of alcohol consumption, other metabolic syndrome components such as abdominal obesity, hypertension, and impaired fasting glucose worsened [41]. A multicenter study in China also revealed that alcohol drinkers exhibited worse lipid profiles in hypertensive and diabetic patients [42]. The significant positive correlation between the TyG index and CVD likelihood in alcohol drinkers can be attributed to the calculation of the TyG index, which is correlated with fasting glucose and fasting triglycerides.

Takeno K et al. found that in non-obese, non-diabetic Japanese men, alcohol consumption showed a significant correlation with decreased hepatic insulin sensitivity and fasting glucose clearance [43]. Chronic alcohol consumption has been reported to disrupt insulin signaling and induce hepatic IR [44]. IR may lead to an increased risk of CVD in both diabetic and non-diabetic populations [32]. IR induces an imbalance in glucose metabolism, leading to hyperglycemia. This, in turn, triggers inflammation and oxidative stress [45]. Additionally, IR can cause systemic lipid disorders, resulting in hypertriglyceridemia. This further induces an increase in the level of free fatty acids, causing an increase in myocardial oxygen consumption [45]. These mechanisms contribute to the increased odds of cardiovascular complications associated with insulin resistance. The chronic hyperglycemic state due to IR induces an increase in glycosylation products, leading to the inactivation of nitric oxide. This impairment of nitric oxide function contributes to endothelial dysfunction, promotes smooth muscle cell proliferation, and results in collagen deposition [18, 46–48]. These metabolic disturbances are not strongly associated with adverse outcomes of CVD. The positive correlation between the TyG index and CVD likelihood in alcohol drinkers can be explained by the contribution of alcohol consumption to the development of IR.

The strength of our study is that it is based on the NHANES database, which utilizes a nationally representative sample of the U.S. population.The sophisticated multi-stage probability sampling methodology used in the NHANES database ensures that the participants in this study accurately represent the non-institutionalized population, which aids in the generalization of results to the U.S. We included 6502 participants in this study, which is a fairly large sample size that ensures strong statistical power and enriches the field. We also analyzed the data using appropriate NHANES sample weights, enhancing the reliability and generalizability of our findings. In addition, we addressed confounding bias by adjusting for a large number of covariates, enhancing the reliability of our results and allowing them to be applied to a wider range of individuals. Finally, we used sensitivity analyses and subgroup analysis models to further enhance the reliability of our assessment regarding the association between the TyG index and the likelihood of CVD.

Despite the strengths of our study, there are potential limitations that should not be overlooked. Firstly, although we adjusted for a number of potentially confounding covariates as much as possible, the possibility of residual confounding remains. Secondly, our analyses were primarily from participants in one country, the United States, and due to factors such as living environment and dietary habits, which may limit the generalizability and applicability of our findings globally, further exploration is essential to validate the generalizability of our main findings across ethnicities and races. Thirdly, the TyG index collected in the NHANES study had only a baseline value, which may have led to an underestimation of the association between the TyG index and the likelihood of CVD due to the lack of the status of the TyG index of the participants over the long term during the follow-up period. Finally, due to the lack of data on fasting insulin levels, it is currently not possible to compare the TyG index with other gold standard indices for evaluating insulin resistance.

## Conclusion

Our findings suggest that the TyG index serve as a valuable tool for predicting the likelihood of CVD development in the general elderly population over 60 years of age in the United States, and the relationship between the TyG index and CVD likelihood is nonlinear. In addition, our study hold significant implications for potentially reducing the cost of screening. These findings are particularly relevant for clinical practice and extensive epidemiological studies. Future studies should explore whether interventions targeting the TyG index may improve clinical prognosis in the elderly population.

### Supplementary Information

**Additional file1: Table S1.** Weighted baseline characteristics of the study population by gender.

**Additional file 2: Table S2.** The association of CVD, stroke, CHF, CHD, heart attack, angina and ASCVD on TyG index levels.

**Additional file 3: Table S3.** The association of CVD and CHD on TyG index levels in different gender subgroup.

## Data Availability

Publicly available datasets were analyzed in this study. This data can be found here: https://www.cdc.gov/nchs/nhanes/index.htm.
