# Supplementary material for: The association between triglyceride-glucose index and the likelihood of cardiovascular disease in the U.S. population of older adults aged ≥ 60 years: a population-based study
Source: Cardiovasc Diabetol. 2024 May 3;23:151. doi: 10.1186/s12933-024-02248-5 (PMC11067197; doi:10.1186/s12933-024-02248-5)
Supplement: Supplementary file 1 — Additional file1: Table S1. Weighted baseline characteristics of the study population by gender. [file 12933_2024_2248_MOESM1_ESM.docx]

**Table S1 Weighted baseline characteristics of the study population by gender**

| TyG index | Female | Male | P value |
| --- | --- | --- | --- |
| Age (year) | 70.20 (0.17) | 69.33 (0.15) | **<0.0001** |
| Serum creatinine (mg/dl) | 0.88 (0.01) | 1.10 (0.01) | **<0.0001** |
| Serum uric acid (umol/L) | 318.47 (1.90) | 364.42 (2.23) | **<0.0001** |
| Total cholesterol (mg/dl) | 204.81 (1.03) | 181.62 (1.20) | **<0.0001** |
| LDL-C (mg/dl) | 116.89 (0.88) | 105.53 (1.05) | **<0.0001** |
| Triglyceride (mg/dl) | 130.13 (1.93) | 131.31 (2.80) | 0.72 |
| Fast glucose (mg/dl) | 111.74 (0.63) | 118.68 (0.96) | **<0.0001** |
| HbA1c (%) | 5.92 (0.02) | 5.98 (0.03) | 0.05 |
| BMI (Kg/m^2) | 29.13 (0.17) | 28.92 (0.17) | 0.34 |
| ACR (mg/g) | 51.25 (5.63) | 62.58 (7.64) | 0.25 |
| eGFR (mL/min/1.73 m^2) | 73.31 (0.40) | 74.03 (0.41) | 0.18 |
| Systolic blood pressure (mmHg) | 131.99 (0.46) | 129.68 (0.55) | **<0.001** |
| Diastolic blood pressure (mmHg) | 65.79 (0.35) | 68.26 (0.36) | **<0.0001** |
| TyG index | 8.73 (0.12) | 8.77 (0.12) | **< 0.0001** |
| Races, % (SE) |  |  | **0.03** |
| Mexican American | 4.07 (0.50) | 3.83 (0.48) |  |
| Non-Hispanic Black | 8.76 (0.64) | 7.26 (0.58) |  |
| Non-Hispanic White | 78.18 (1.17) | 79.80 (1.22) |  |
| Others | 8.99 (0.69) | 9.11 (0.78) |  |
| Educational levels, % (SE) |  |  | **< 0.0001** |
| Less than 9th grade | 9.05 (0.61) | 9.36 (0.62) |  |
| 9-11th grade | 11.40 (0.72) | 10.37 (0.86) |  |
| High school graduate | 27.56 (1.09) | 23.06 (1.14) |  |
| Some college or AA degree | 29.14 (1.23) | 25.84 (1.28) |  |
| College graduate or above | 22.85 (1.21) | 31.37 (1.59) |  |
| PIR ,% (SE) |  |  | **< 0.0001** |
| <1 | 10.62 (0.75) | 7.90 (0.64) |  |
| 1-4 | 58.16 (1.51) | 52.58 (1.45) |  |
| >4 | 31.22 (1.61) | 39.52 (1.51) |  |
| BMI, % (SE) |  |  | **<0.0001** |
| Normal weight | 28.73 (1.14) | 23.87 (1.09) |  |
| Overweight | 32.64 (1.12) | 40.10 (1.31) |  |
| Obesity | 32.64 (1.12) | 41.31 (2.04) |  |
| Smoke, % (SE) |  |  | **< 0.0001** |
| Never | 59.12 (1.29) | 35.30 (1.35) |  |
| Former | 30.80 (1.22) | 52.17 (1.22) |  |
| Now | 10.08 (0.78) | 12.53 (0.87) |  |
| Alcohol use, % (SE) | 70.43 (1.07) | 70.01 (1.23) | 0.78 |
| Hypertension, % (SE) | 67.91 (1.13) | 65.50 (1.20) | 0.09 |
| DM, % (SE) | 29.24 (1.04) | 34.63 (1.45) | **0.002** |
| CVD, % (SE) | 19.42 (1.07) | 30.20 (1.17) | **< 0.0001** |
| Stroke, % (SE) | 7.88 (0.59) | 8.14 (0.71) | 0.79 |
| CHD, % (SE) | 6.38 (0.58) | 15.85 (0.94) | **< 0.0001** |
| CHF, % (SE) | 6.03 (0.51) | 8.14 (0.72) | **0.02** |
| ASCVD, % (SE) | 17.17 (0.98) | 28.55 (1.16) | **< 0.0001** |
| Heart attack, % (SE) | 5.91 (0.53) | 14.36(0.84) | **< 0.0001** |
| Angina, % (SE) | 4.52 (0.45) | 8.14 (0.71) | **< 0.0001** |

LDL-C: low-density lipoprotein cholesterol; HDL-C: high-density lipoprotein cholesterol; ACR: urinary albumin: creatinine ratio; eGFR:estimated-glomerular filtration rate; BMI: body mass index; PIR: family income-poverty ratio; DM: diabetes; CHF: congestive heart failure; CVD: cardiovascular disease; CHD:congestive heart disease; ASCVD: atherosclerotic cardiovascular disease.

|  |
| --- |
