# Supplementary material for: The association between triglyceride-glucose index and the likelihood of cardiovascular disease in the U.S. population of older adults aged ≥ 60 years: a population-based study
Source: Cardiovasc Diabetol. 2024 May 3;23:151. doi: 10.1186/s12933-024-02248-5 (PMC11067197; doi:10.1186/s12933-024-02248-5)
Supplement: Supplementary file 2 — Additional file 2: Table S2. The association of CVD, stroke, CHF, CHD, heart attack, angina and ASCVD on TyG index levels. [file 12933_2024_2248_MOESM2_ESM.docx]

**Additional file 2: Table S2 The association of CVD, stroke, CHF, CHD, heart attack, angina and ASCVD on TyG index levels**

| TyG index | β (95%CI) |  |  |
| --- | --- | --- | --- |
|  | Model 1 | Model 2 | Model 3 |
| CVD | 0.16 (0.10, 0.21), **p<0.0001** | 0.17 (0.11, 0.22),  **p<0.0001** | 0.05 (0.01, 0.09)  **p=0.02** |
|  |  |  |  |
| Stroke | 0.09 (0.02, 0.16),  **p=0.01** | 0.1 (0.03, 0.17),  **p=0.005** | -0.01 (-0.08, 0.06),  p=0.78 |
|  |  |  |  |
| CHF | 0.17 (0.09, 0.25), **p<0.0001** | 0.18 (0.10, 0.26),  **p<0.0001** | 0.03 (-0.04, 0.10),  p=0.41 |
|  |  |  |  |
| CHD | 0.15 (0.08, 0.23),  **p<0.001** | 0.15 (0.07, 0.22),  **p<0.001** | 0.07 (0.01, 0.13),  **p<0.001** |
|  |  |  |  |
| Heart attack | 0.12 (0.05, 0.19),  **p=0.001** | 0.12 (0.04, 0.19),  **p=0.002** | 0.02 (-0.03, 0.08),  p=0.44 |
|  |  |  |  |
| Angina | 0.21 (0.12, 0.31), **p<0.0001** | 0.21 (0.11, 0.31),  **p<0.0001** | 0.06 (-0.02, 0.14)  p=0.17 |
|  |  |  |  |
| ASCVD | 0.15 (0.09, 0.20), **p<0.0001** | 0.15 (0.10, 0.21),  **p<0.0001** | 0.04 (-0.01, 0.08)  p=0.11 |

95%CI: 95% Confidence Interval

Model 1: No covariates were adjusted.

Model 2: Age, gender, and race were adjusted.

Model 3: Age, gender, race, education level, PIR, BMI, serum creatinine, serum uric acid, total cholesterol, HbA1c, ACR, eGFR, systolic blood pressure, diastolic blood pressure, DM, hypertension, smoking and alcohol consumption status were adjusted.
