# Supplementary material for: The association between triglyceride-glucose index and the likelihood of cardiovascular disease in the U.S. population of older adults aged ≥ 60 years: a population-based study
Source: Cardiovasc Diabetol. 2024 May 3;23:151. doi: 10.1186/s12933-024-02248-5 (PMC11067197; doi:10.1186/s12933-024-02248-5)
Supplement: Supplementary file 3 — Additional file 3: Table S3. The association of CVD and CHD on TyG index levels in different gender subgroup. [file 12933_2024_2248_MOESM3_ESM.docx]

**Additional file 3: Table S3 The association of CVD and CHD on TyG index levels in different gender subgroup.**

| TyG index | β  (95%CI) | |  | |  |
| --- | --- | --- | --- | --- | --- |
|  |  | | P for interaction | |  |
| CVD |  |  | |  | |
| **Gender** |  | **0.047** | |  | |
| Female | -0.012  (-0.076, 0.052), p=0.717 |  | |  | |
| Male | 0.116  (0.049, 0.182),  **p<0.001** |  | |  | |
|  |  |  | |  | |
| CHD  **Gender** |  | 0.286 | |  | |
| Female | 0.013  (-0.103, 0.129),  p=0.830 |  | |  | |
|  |  |  | |  | |
| Male | 0.129  (0.049, 0.208),  **p=0.002** |  | |  | |
|  |  |  | |  | |
|  |  |  | |  | |
|  |  |  | |  | |
|  |  |  | |  | |
